# Experimental evaluation of pathogenicity and acquired immunity of *Eimeria* species, *E. uekii* and *E. raichoi*, infecting Japanese rock ptarmigans in a subspecies of the birds

**DOI:** 10.1016/j.ijppaw.2023.09.005

**Published:** 2023-09-09

**Authors:** Makoto Matsubayashi, Moemi Kinoshita, Sayaka Tsuchida, Atsushi Kobayashi, Naoya Tamura, Tomoyuki Shibahara, Yasutoshi Kido, Akira Kaneko, Kazumi Sasai, Kazunari Ushida

**Affiliations:** a Graduate School of Veterinary Medical Sciences, Osaka Metropolitan University, Osaka, 598-8531, Japan; b Department of Veterinary Parasitology, Faculty of Veterinary Medicine, Airlangga University, Surabaya, 60115, Indonesia; c College of Bioscience and Biotechnology, Chubu University, Aichi, 487-8501, Japan; d Shin-etsu Nature Conservation Office, Ministry of the Environment, Ministry of Environment, Nagano, 380-0846, Japan; e Nagano Chausuyama Zoo, Nagano, 388-8016, Japan; f Division of Pathology and Pathophysiology, National Institute of Animal Health, National Agriculture and Food Research Organization, Ibaraki, 305-0856, Japan; g Departments of Virology and Parasitology, Graduate School of Medicine, Osaka Metropolitan University, Osaka, 545-8585, Japan; h Osaka International Research Center for Infectious Diseases, Osaka Metropolitan University, Osaka, 545 -8585, Japan

**Keywords:** *Eimeria raichoi*, *Eimeria uekii*, Japanese rock ptarmigan, Pathogenicity

## Abstract

Japanese rock ptarmigans (*Lagopus muta japonica*) are birds that inhabit only alpine regions of central Honshu Island, Japan, known as the Japanese Alps. The number of these birds has recently declined, and *in situ* and *ex situ* national conservation programs for Japanese rock ptarmigans have been initiated. The infections of *Eimeria* spp. as protozoan parasites of the phylum Apicomplexa, *E. uekii* and *E. raichoi*, were frequently reported in the birds. However, the virulence of these *Eimeria* parasites has not been determined. Here, we analyzed the pathogenicity of these *Eimeria* parasites using experimental infections of a subspecies model of Japanese rock ptarmigans, Svalbard rock ptarmigans (*Lagopus mutus hyperboreus*), and evaluated acquired protective immunity against challenge in birds tolerant of low-dose inoculation with *Eimeria* parasites. Following inoculation with two *Eimeria* parasites derived from Japanese rock ptarmigans (dose range of 4 × 10^4^ to 4 × 10^2^ for *E. uekii* and 1.7 × 10^4^ to 4 × 10^1^ for *E. raichoi*), oocysts were detected at 6–8 days post-inoculation (PI), and the maximum number of oocysts per gram of feces was observed 7–10 days PI and then gradually decreased. The mortality rate and reduction in weight gain of chicks increased following high-dose inoculation of oocysts with abnormal feces (soft and diarrhea). Developmental zoites were detected histopathologically in epithelial tissues and sometimes the lamina propria from the duodenum to the colon. Chicks that survived low-dose inoculation did not show clear clinical symptoms after challenge inoculation. Our results suggest that the pathological characteristics of *Eimeria* parasites infecting Japanese rock ptarmigans include abnormal feces and reduction in weight gain, resulting in mortality in cases of heavy infection due to high-dose inoculation. These findings provide helpful data for Japanese rock ptarmigan conservation efforts.

## Highlights

• Infection with two *Eimeria* spp. has been frequently reported in Japanese rock ptarmigans.
• Pathological characteristics can be abnormal feces and reduction in weight gain, and mortality in heavy infection.
• Immunity acquired by low-dose infection gives tolerance to the challenge inoculation.

## Graphical abstract

**Figure:**
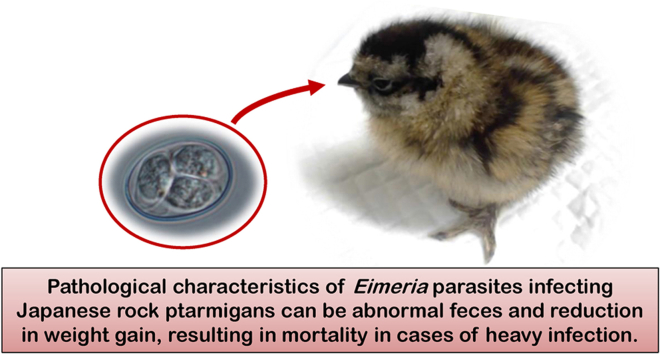

## 1 Introduction

The Japanese rock ptarmigan (*Lagopus muta japonica*) is a bird species belonging to the order Galliformes, family Phasianidae, and genus *Lagopus* and inhabits only the high mountainous regions of Honshu Island, Japan, known as the Japanese Alps. The species was designated a Special Natural Monument of Japan in 1955; however, the number of the birds has decreased, and the population is now estimated at less than 2000 (Ministry of Environment Japan, 2012). Therefore, the Japanese rock ptarmigan has been listed in the EN (endangered) category of the Japanese Red List (Ministry of the Environment Japan, 2020), and both *in situ* and *ex situ* national conservation programs for Japanese rock ptarmigans have been initiated (Ministry of Environment Japan, 2012).

Infection with two *Eimeria* spp., *E. uekii* and *E. raichoi* (previously referred to as types A and B, respectively), protozoan parasites belonging to the phylum Apicomplexa, has been frequently reported in Japanese rock ptarmigans (Kamimura and Kodama, 1981; Ishihara et al., 2006; Matsubayashi et al., 2018a, Matsubayashi et al., 2018b). During recent cage protection efforts for a family of Japanese rock ptarmigans as part of an *in situ* conservation program, histopathological analyses of dead chicks revealed evidence of severe infections with these parasites in the intestinal mucosa (Matsubayashi et al., 2020, Matsubayashi et al., 2021). These findings suggest the parasites adversely affect the health of the birds; however, details regarding the virulence of the parasites, including clinical symptoms, remain unknown.

In general, the *Eimeria* spp. are highly host specific and cause enteric diseases such as coccidiosis, which is characterized by bloody feces or severe diarrhea and eventual death. *Eimeria* spp. infections in chickens (order Galliformes and family Phasianidae) cause significant economic losses in the poultry industry (Hafez, 2008; Tewari and Maharana, 2011). However, not all species of *Eimeria* are highly pathogenic, as some species of *Eimeria* parasites (e.g., *E. acervulina* and *E. maxima*) cause watery diarrhea and/or reduced weight gain as chronic symptoms, whereas others (e.g., *E. tenella* and *E. necatrix*) cause bloody feces resulting in high mortality (Morris et al., 2007; Mesa-Pineda et al., 2021). Previously, phylogenetic analyses suggested that *E. uekii* was genetically closely related to *Eimeria* spp. that affect chickens, whereas *E. raichoi* was related to species affecting turkeys, which were in the same family and subfamily as Japanese rock ptarmigans, respectively (Matsubayashi et al., 2018a). However, whether the pathogenicity of *Eimeria* infections in chickens or turkeys and rock ptarmigans is similar has not been determined; thus, data regarding the virulence of the two *Eimeria* spp. in Japanese rock ptarmigans are needed for conservation programs.

In our previous study, we experimentally evaluated the infectivity of parasites derived from Japanese rock ptarmigans using other bird species. We found that *E. uekii* failed to infect any of the examined birds, including chickens, and *E. raichoi* could be successfully cross-transmitted only to turkeys, without any clinical symptoms (Matsubayashi et al., 2023). Thus, in the present study, the infectivity of the parasites was assessed using Svalbard rock ptarmigans (*Lagopus mutus hyperboreus*) as a subspecies of Japanese rock ptarmigans, and the virulence of the parasites was analyzed using a more closely related animal model in the same genus. Furthermore, birds tolerant to low-dose challenge with *Eimeria* parasites were challenged at the virulence dose, and the acquired protective immunity against challenge inoculation was evaluated.

## 2 Materials and methods

### 2.1 Parasites

Fecal samples from wild Japanese rock ptarmigans, including the family of wild birds protected in cages as part of an *in situ* conservation program (Kobayashi et al., 2019; Matsubayashi et al., 2020), were collected at Mt. Kitadake, Mt. Tateyama, and Mt. Norikuradake from June to August in 2018 and 2020. The feces were examined by the sugar floatation centrifuge method, as previously reported (Matsubayashi et al., 2018b; Ekawasti et al., 2019), and oocysts of *E. uekii* and *E. raichoi* were purified using positive fecal samples by the sugar floatation centrifuge method, as previously reported (Matsubayashi et al., 2020). After sporulation in approximately 1% potassium dichromate solution (Nacalai Tesque, Kyoto, Japan) at 27–28 °C for several days, the oocysts were stored at 4 °C for 1–2 months until use for experimental infections. Before experimental inoculation, the oocysts were treated with 5–10% sodium hypochlorite (Nacalai Tesque) for 5 min at 4 °C and washed with phosphate-buffered saline (PBS, pH 7.4).

### 2.2 Birds

For experimental infections, Svalbard rock ptarmigans (*L. muta hyperboreus*) were used. The eggs of the birds were kindly supplied by Nagano Chausuyama Zoo (Nagano, Japan) and hatched in incubators (Belbirds, Chiba, Tokyo) at Osaka Metropolitan University. All animals used in the present study were handled in accordance with the guidelines and policies for animal studies of the Osaka Metropolitan University, and all experiments were approved by the Animal Care and Use Committee of the Osaka Metropolitan University (approval numbers 15003 and 21022).

### 2.3 Experimental infections

By the fecal examinations, all of the positive fecal samples were found to involve mixed infection with *E. uekii* and *E. raichoi*. Because we could not isolate only the oocysts of single species of *Eimeria* parasites, *E. uekii* or *E. raichoi*, the inoculation dose of the oocysts was prepared based on the number of *E. uekii* as the initial analysis to assess their pathogenicity. Namely, Svalbard rock ptarmigan chicks were inoculated with oocysts diluted in 100–200 μl of PBS at 4–6 days of age via oral administration. The inoculation doses of *E. uekii* were 4 × 10^4^ (for 5 birds), 2 × 10^4^ (for 3 birds), 4 × 10^3^ (for 4 birds), and 4 × 10^2^ (for 4 birds), which contained 1.7 × 10^4^ to 2 × 10^1^ oocysts of *E. raichoi* (Table 1). Additionally, two Svalbard rock ptarmigans were inoculated with 4 × 10^4^ *E. raichoi* oocysts that had been passaged in turkeys and purified using the same method (Matsubayashi et al., 2023). As a control, 4 birds were orally administered the same volume of PBS.

**Table 1:** Summary of experimental inoculation of Svalbard rock ptarmigans.

Number of oocysts inoculated		No. of surviving birds at 14 days post-inoculation/No. of examined birds	Average weight gain (g) at 14 days (no. of birds)	Notes
*E. uekii*	*E. raichoi*			
4 × 10^4^	17 × 10^3^ or 2 × 10^3^	2/5 (40.0%)	57.2 (n = 2)	Soft stool/diarrhea
2 × 10^4^	8 × 10^3^	2/3 (66.7%)	49.0 (n = 2)	Soft stool/diarrhea
4 × 10^3^	17 × 10^2^ or 2 × 10^2^	3/4 (75.0%)	97.5 (n = 2)	Active behavior
4 × 10^2^	17 × 10^1^ or 2 × 10^1^	3/4 (75.0%)	98.1 (n = 3)	Active behavior
–	4 × 10^4^	0/2 (0%)	–	Severe leg deformities
PBS (Control)		2/4 (50.0%)	83.0 (n = 2)	Active behavior

Feces were collected every 1–2 days after inoculation with oocysts, from 4 days until 14 days. The collected feces were pooled as samples from 1 to 3 examined birds, mixed thoroughly, and then examined using the sugar floatation centrifuge method, as described above. For the number of oocysts per gram of feces (OPG), 1 g of feces was diluted with 5 ml of water, and 8 μl of the diluted sample was placed on a Plankton counting chamber (Rigo, Tokyo, Japan), after which the number of oocysts was determined. In the case few oocysts were obtained, the oocysts were purified using the entire fecal sample, as described above, and the number was determined by counting the oocysts in the chambers. The birds were weighed, and their clinical symptoms were recorded until 14 days post-inoculation (PI). During monitoring, all birds were administered oxytetracycline hydrochloride (Kyoritsu Seiyaku, Tokyo, Japan) in the drinking water, according to the manufacturer's instructions.

### 2.4 Histopathological analyses

For histopathological examinations, birds in the groups inoculated with 4 × 10^4^ *E. uekii* (total of 5 birds each at 2, 4, 5, 10, and 37 days PI), 4 × 10^3^ *E. uekii* (1 bird at 4 days PI), or 4 × 10^2^ *E. uekii* (3 birds each at 6, 16, and 24 days PI), and *E. raichoi* (ranging 17 × 10^3^ to 17 × 10^1^), were examined (Table 2). The experimental infection was described in the previous sentence. Unexpectedly, some of these birds were found dead during the intervals between observations, and the others were euthanized according to the animal care guidelines of Osaka Metropolitan University.

**Table 2:** Histopathological analysis of developmental parasites in birds after inoculation with *Eimeria* spp.

Inoculation doses									
*E. uekii*	4 × 10^4^					4 × 10^3^	4 × 10^2^		
*E. raichoi*	17 × 10^3^ or 2 × 10^3^					17 × 10^2^	17 × 10^1^		
Days post inoculation	2	4	5	10	37	4	6	16	24
Glandular stomach	–	–	–	–	–	–	–	–	–
Muscular stomach	–	–	–	–	–	–	–	–	–
Duodenum	–	+	+	–	–	+	–	–	–
Jejunum	+	+	++	–	–	–	–	–	–
Ileum	–	+++	++	+	+	+	–	+	–
Cecum	–	+	–	–	–	–	–	–	–
Colon	–	++	–	–	–	–	–	–	–
other organs*	–	–	–	–	–	–	–	–	–

(−, no parasites; +, a few parasites in the intestinal mucosa in some fields; ++, 1–5 parasites in the intestinal mucosa per field; +++, >5 parasites per field).

*; heart, liver, kidneys, lungs, brain, and thoracic skeletal muscles.

The heart, liver, kidneys, lungs, brain, thoracic skeletal muscles, muscular stomach, glandular stomach, duodenum, jejunum, ileum, cecum, and colon were removed from each chick. The organs were fixed in 10% neutral-buffered formalin (Nacalai Tesque) for several weeks and processed for routine histological examinations. Tissue sections were stained with hematoxylin and eosin. Stained histological sections were examined under a light microscope (200 × or 400 × ). Histological scores were determined based on the number of parasites (−, no parasites; +, a few parasites in some fields of the intestinal mucosa; ++, 1–5 parasites per field in the intestinal mucosa; +++, >5 parasites per field).

Immunohistochemical examinations were conducted to confirm the parasites, as previously reported (Iwanaga et al., 2022; Matsubayashi et al., 2023). Briefly, paraffin-embedded sections of the tissues were incubated with 3% hydrogen peroxide, and antigen was retrieved using 0.1% actinase E solution in PBS. After blocking with 10% normal goat serum, sections were reacted with rabbit anti–*Eimeria tenella* antibody (Matsubayashi et al., 2012). The sections were then incubated with the secondary antibody (Histofine Simple Stain MAX-PO Multi; Nichirei Bioscience Inc., Tokyo, Japan) and treated with aminoethyl carbazole substrate solution (Histofine Simple Stain AEC solution; Nichirei Bioscience). Finally, the sections were counterstained with hematoxylin.

### 2.5 Evaluation for acquired immunity

To evaluate acquired immunity against *Eimeria* parasites resulting from primary infection with a low dose of oocysts, challenge infections were designed as described below. As the primary inoculation, 2 birds in the inoculation group were inoculated with 4 × 10^3^ *E. uekii* (including 2 × 10^2^ oocysts of *E. raichoi*), and 1 bird was inoculated with 4 × 10^2^ *E. uekii* (including 2 × 10^1^ oocysts of *E. raichoi*). The birds of the inoculation groups survived without any clinical symptoms following experimental infection, as described above. For the PBS group, 2 birds were treated with PBS. At 26–27 days PI in the primary examinations, birds were orally injected with 4 × 10^4^ *E. uekii* and 2 × 10^3^ *E. raichoi* for 5 days as challenge inoculations. The birds were weighed, and their clinical signs were observed. Except for 1 bird that died suddenly, the remaining birds were euthanized at 7 days after challenge according to the animal care guideline of the Osaka Metropolitan University and then histopathologically analyzed as described above.

## 3 Results

**Fig. 1:**
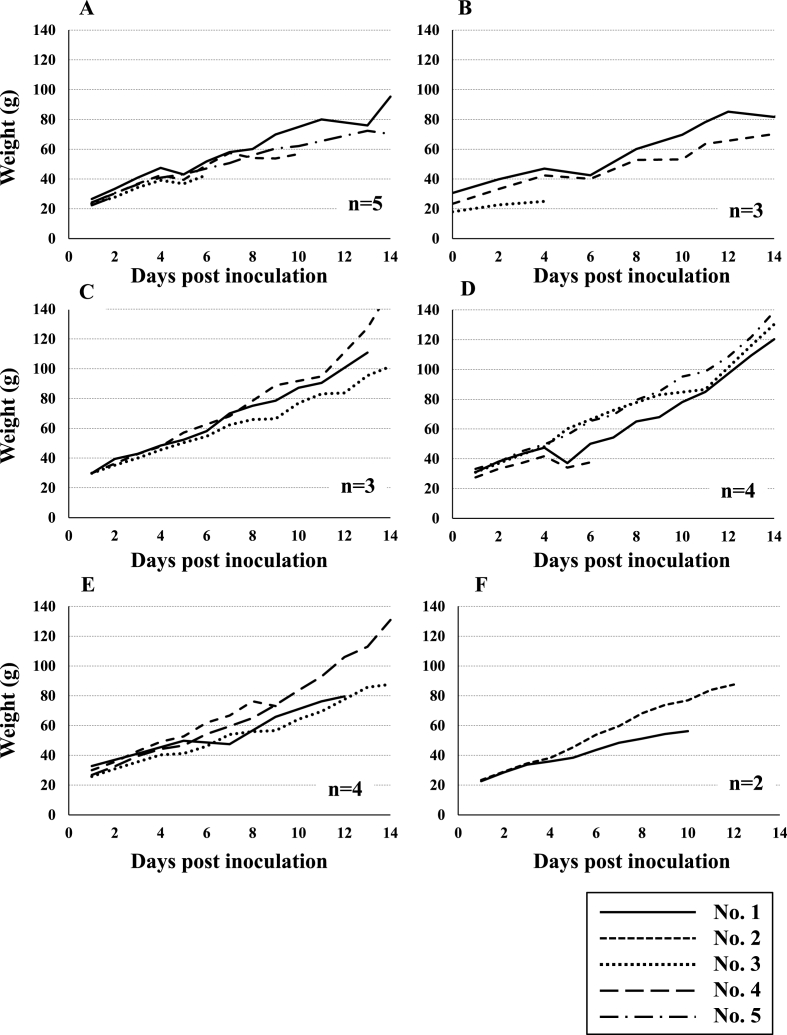
Weight gain of Svalbard rock ptarmigans after inoculation with *E. uekii* and *E. raichoi* oocysts. The inoculation doses of *E. uekii* and *E. raichoi* were 4 × 10^4^ and 17 × 10^3^ or 4 × 10^3^ (A), 2 × 10^4^ and 8 × 10^3^ (B), 4 × 10^3^ and 17 or 4 × 10^2^ (C), and 4 × 10^2^ and 17 or 4 × 10^1^ (D), respectively. Group E was administered PBS as a control, and group F was inoculated with 4 × 10^4^ oocysts of *E. raichoi* that had been passed in turkeys. “n” indicates the numbers of examined chicks.

**Fig. 2:**
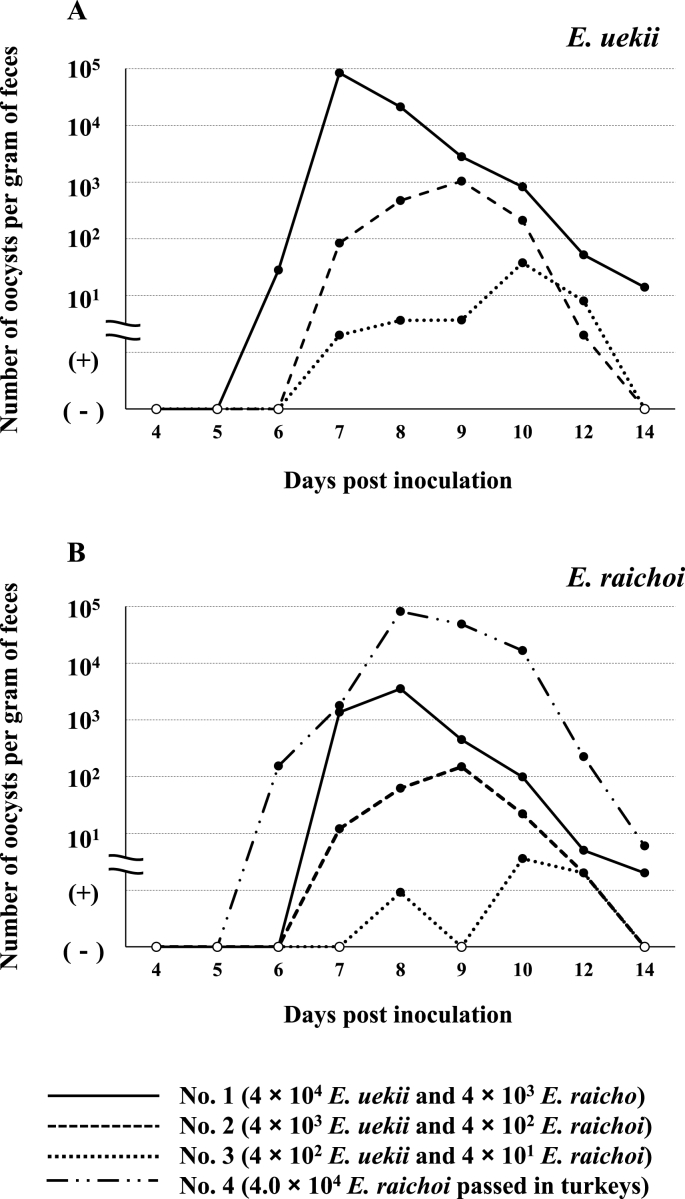
The number of oocysts per gram of feces (OPG) after inoculation with *E. uekii* and *E. raichoi* oocysts. Panels A and B show *E. uekii* and *E. raichoi* OPG, respectively. Chicks Nos. 1–3 were inoculated depending on the manner of oocysts *E. uekii* and *E. raichoi* as described at the bottom of Figure, and mouse no. 4 was inoculated with oocysts of *E. raichoi*.

The results, including mortality rate and weight gain during experimental infection of Svalbard rock ptarmigans, are summarized in Table 1 and shown in Fig. 1. Rearing rock ptarmigans is generally difficult, and thus, some chicks died of unknown cause. In the present study, a few chicks died even in the groups receiving lower doses of *E. uekii* and PBS. Additionally, 2 chicks in the group inoculated with 4 × 10^4^ *E. raichoi* showed severe leg deformities after inoculation and were euthanized according to the guideline of the Osaka Metropolitan University. Consequently, the survival percentage tended to decrease depending on the number of inoculated oocysts. The average weight gain in the group receiving >10^4^ oocysts of *E. uekii* (and *E. raichoi*) was almost half of that of chicks receiving <10^3^ oocysts of *E. uekii* (and *E. raichoi*) and of the PBS group until 14 days PI. Some chicks in the group receiving >10^4^ oocysts of *E. uekii* (and *E. raichoi*) exhibited soft stool or diarrhea by 4–6 days PI. In most of the chicks, the body weight began to decrease 4–6 days PI (Fig. 1). Oocysts were detected at 6–8 days PI, and the maximum OPG was found at 7–10 days PI, and the day of maximum OPG tended to be delayed depending on the oocyst inoculation dose (Fig. 2). The OPG then gradually degreased, and some groups became negative for the presence of oocysts.

**Fig. 3:**
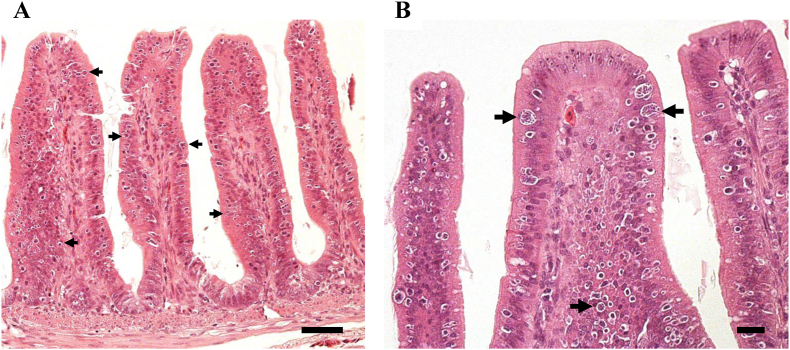
Histopathological photomicrographs of sections from an experimentally inoculated chick (Svalbard rock ptarmigan). Panel A shows the ileum of the chick at 4 days PI after inoculation with 4 × 10^4^ *E. uekii* and 2 × 10^3^ *E. raichoi* oocysts. Panel B is a higher magnification of the section. Arrows indicate developmental zoites or mature schizonts (in panel B). Scale bars in panels A and B are 20 μm and 50 μm, respectively.

In histopathological analyses, developmental zoites of *Eimeria* parasites were detected from the duodenum to colon of the chicks (Table 2). In the group inoculated with 4 × 10^4^ *E. uekii* and 17-2 × 10^3^ *E. raichoi* oocysts, numerous developmental zoites (mainly schizonts and trophozoites) were seen in the epithelial cells and sometimes the lamina propria at 4 and 5 days PI (Fig. 3). The number of these zoites then decreased between 10 and 37 days PI, although not all of the zoites disappeared. Although we could not determine detailed pathological changes that occurred in some chicks due to the degraded state of the organs after sudden death, clear signs of an immune response, including infiltration of inflammatory cells, were rarely observed. In the groups receiving a lower dose (4 × 10^3^ *E. uekii* and 17 × 10^2^ *E. raichoi* or 4 × 10^2^ *E. uekii* and 17 × 10^1^ *E. raichoi*), fewer or no developmental zoites were detected in the histopathological analyses. The other organs were found to be negative in all examined chicks. The developmental zoites detected at each stage were morphologically similar, and thus, the species of *Eimeria* present in the lesions could not be identified.

**Fig. 4:**
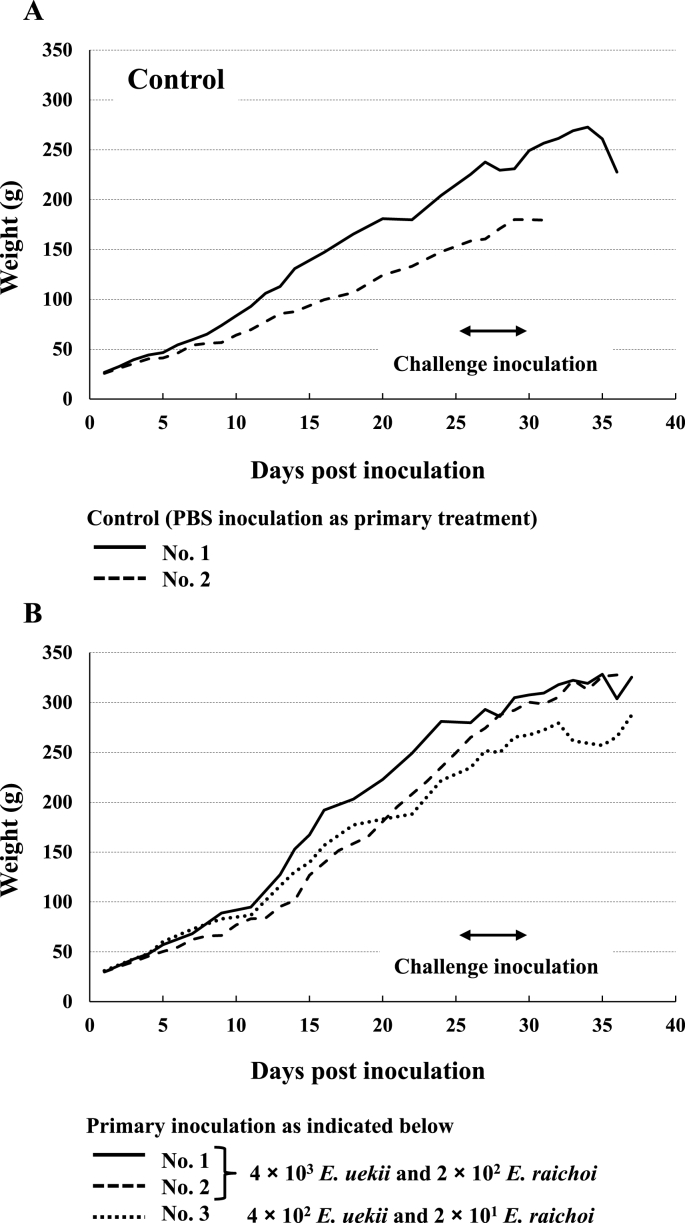
Weight gain of Svalbard rock ptarmigans after challenge inoculation. Panels A and B show the weight of control (PBS) and primary inoculated chicks, respectively. Two-direction arrows indicate the period of challenge inoculation, 4 × 10^4^ *E. uekii* and 2 × 10^3^ *E. raichoi* for 5 days.

**Fig. 5:**
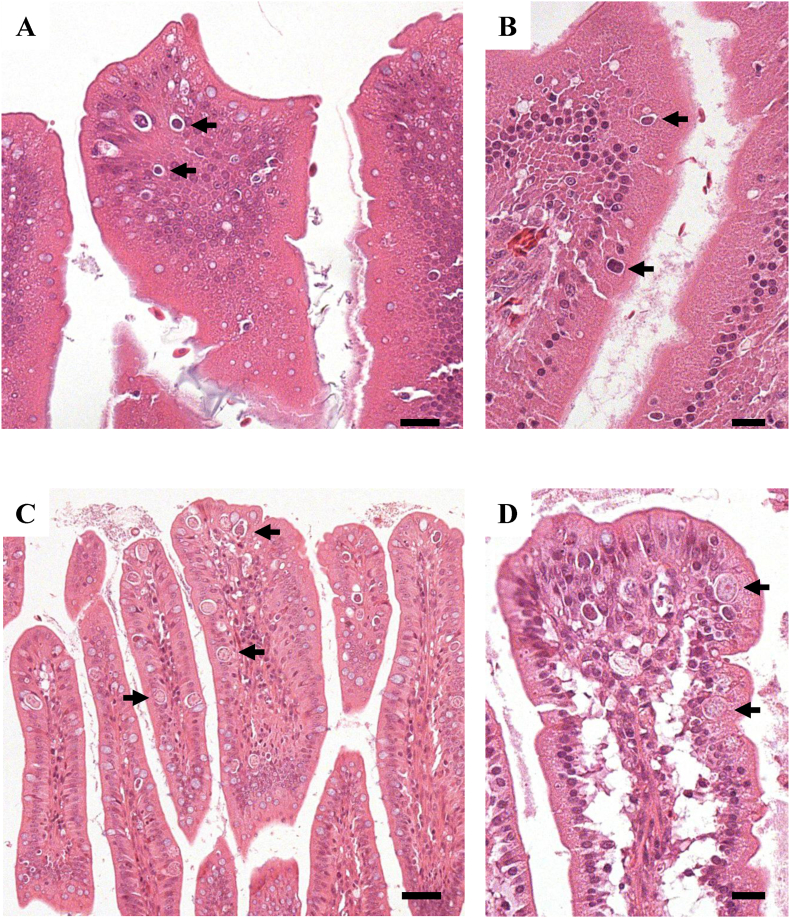
Histopathological photomicrographs of sections of the intestines of challenged chicks. Panels A and B show the ileum and duodenum of the primary inoculated chick at 7 days PI, respectively. Panels C and D show the ileum and colon of a non-inoculated chick at 7 days PI, respectively. Arrows indicate developmental zoites (sexual stages in panels C and D). Scale bars are 20 μm in panels A and C and 50 μm in panels B and D.

Some chicks in the lower-dose experimental infection groups (4 × 10^3^ or 4 × 10^2^ *E. uekii* and *E. raichoi*) survived without any clinical symptoms (Table 1). Therefore, a total of 3 birds were challenged by injection with 4 × 10^4^ *E. uekii* and 2.6 × 10^3^ *E. raichoi* for 5 days, and 2 birds were injected with PBS as a control. The chicks which had been inoculated with a lower dose of oocysts survived the challenge (Table 3). However, 2 primary non-infected chicks (PBS control group) showed softened stool or diarrhea in addition to weight decrease after the challenge infection (Fig. 4), resulting in death. Histopathological analyses revealed the presence of developmental zoites from the duodenum to the colon in primary infected and non-infected (PBS group) chicks; however, relatively more zoites were detected in the chicks of the latter group (Table 4). At 7 days after challenge, most of the zoites detected in the primary infected groups were small and immature schizonts parasitizing the epithelial cells and often the lamina propria (Fig. 5A and B). In non-infected chicks (PBS group), many mature zoites were found in sexual stages, as micro- and macro-gametocytes and as zygotes in the more-developed stages (Fig. 5C and D). Histopathological analyses indicated absent or minimal immune response in chicks in the primary infected groups, but pathological findings in non-infected chicks (PBS group) could not be determined due to the degraded state of the organs after death.

**Table 3:** Challenge inoculation of Svalbard rock ptarmigans.

Primary injection		Secondary injection for 5 days		No. surviving at 14 days post-inoculation/No. of examined birds	Average weight gain (g) until 1 day after secondary injection (no. of birds)	Notes
*E. uekii*	*E. raichoi*	*E. uekii*	*E. raichoi*			
4 × 10^3^	17 × 10^2^ or 2 × 10^2^	4 × 10^4^	2.6 × 10^3^	2/2 (100%)	290.0 (n = 2)	Active behavior
4 × 10^2^	2 × 10^1^	4 × 10^4^	2.6 × 10^3^	1/1 (100%)	241.4 (n = 1)	Active behavior
− (PBS)		4 × 10^4^	2.6 × 10^3^	0/2 (0%)	218.1 (n = 2)	soft stool/diarrhea

**Table 4:** Histopathological analysis of developmental parasites in birds after challenge injection.

Primary inoculation	*E. uekii*	4 × 10^3^		4 × 10^2^	− (PBS)
	*E. raichoi*	17 or 2 × 10^2^		2 × 10^1^	
Secondary inoculation	*E. uekii*	4 × 10^4^		4 × 10^4^	4 × 10^4^
	*E. raichoi*	2.6 × 10^3^		2.6 × 10^3^	2.6 × 10^3^
Glandular stomach		–	–	–	–
Muscular stomach		–	–	–	–
Duodenum		+++	+	++	++
Jejunum		+	+	+	++
Ileum		++	+	+	+++
Cecum		–	–	–	+
Colon		+	–	+	+++
Other organs*		–	–	–	–

Days after secondary injection	7 days				

(−, no parasites; +, a few parasites in the intestinal mucosa in some fields; ++,1–5 parasites in the intestinal mucosa per field; +++, >5 parasites per field).

*; heart, liver, kidneys, lungs, brain, and thoracic skeletal muscles.

## 4 Discussion

Previous studies have reported a high prevalence of *E. uekii* and *E. raichoi* in wild Japanese rock ptarmigans (Kamimura and Kodama, 1981; Ishihara et al., 2006; Matsubayashi et al., 2018a, Matsubayashi et al., 2018b). In the present study, the pathogenicity of two different *Eimeria* spp. was evaluated using subspecies of the birds as an experimental model although the oocysts were prepared as mixed *Eimeria* species due to the difficulties for the isolation of the single species. Depending on the inoculated dose of oocysts, chicks shed abnormal (but not bloody) feces and weight gain decreased, and some chicks died during monitoring. Thus, the pathological characteristics of the parasites appears to be chronic or subacute, and clinical symptoms can appear if the birds ingest >10^4^ oocysts of *E. uekii* and >10^3^ oocysts of *E. raichoi* at least, although affections on pathogenicity by the mixed infections remain unknown.

Clinical signs resulting from experimental inoculation were observed 4–6 days PI before oocysts were shed in feces, and large numbers of developmental parasites were confirmed in the epithelial tissue and sometimes the lamina propria of the intestines at 4 and 5 days PI. In chickens infected with *Eimeria* spp., the degree of pathogenicity is associated with the parasitized regions and size of the developmental parasites, especially in the asexual stages. For example, the second-generation schizonts of *E. tenella* develop in the lamina propria and grow to >35 μm in size and disrupt the mucosum, resulting in bloody feces (Matsubayashi et al., 2019, Matsubayashi et al., 2012). The size of schizonts of *Eimeria* spp. causing chronic infection (e.g., *E. acervulina* and *E. praecox*) was approximately 10 μm, and these schizonts mainly parasitize epithelial cells (Shirley et al., 1984; McDonald et al., 1982). Although we could not identify the *Eimeria* spp. infecting Japanese rock ptarmigans via histopathological analyses, the schizonts of the parasites were approximately 10 μm in diameter and primarily infected epithelial cells. Thus, the parasites did not cause a high rate of mortality but did induce gut-barrier dysfunction and thereby directly or indirectly affected the health of the birds by causing diarrhea and a reduction in weight gain, especially following high-dose inoculation of oocysts. Although details regarding the transmission routes of *Eimeria* parasites and degree of infection (e.g., number of infecting oocysts) in wild ptarmigans in the Japanese Alps remain unknown, exposure to high doses of the parasites might lead to a decline in the population. Additionally, to breed potentially infected birds from the wild in institutions such as zoos, it is necessary to control or treat any possible infection in the birds with a coccidiostat.

We also inoculated chicks that could tolerate infection with a low dose (approximately <10^4^ oocysts of *E. uekii* and <10^3^ oocysts of *E. raichoi*) with the virulent dose of oocysts (>10^4^ oocysts of *E. uekii* and >10^3^ oocysts of *E. raichoi*). These chicks survived the challenge inoculation, unlike control birds. Histopathological analyses showed that most of the developmental zoites were immature or could have stopped developing before the sexual stage, and the number of zoites was relatively small compared with the control. The development of *Eimeria* spp. zoites in primary immunized mice was shown to stop at the second of 4 asexual generation stages among a total of 5 stages (4 asexual and 1 sexual) after challenge (Ono et al., 2016). Although we could not determine the OPG after challenge, the number of shed oocysts might be small. These results indicate that immunity acquired following infection with a low dose of oocysts could play an essential role in protecting against subsequent heavy challenge by inhibiting the development of the parasites. Although details the infection route remain to be clarified, it could be hypothesized that chicks directly or indirectly ingest the oocysts in feces shed by infected parent birds and thereby develop immunity. This acquired immunity might be essential to ensure survive in of birds in the wild and furthermore could be necessary for generating protective immunity against *Eimeria* parasites in artificially raised birds released into natural habitats.

Details regarding *Eimeria* infections in Japanese rock ptarmigans are poorly understood. Generally, in other animals, the number of *Eimeria* spp. oocysts shed in the feces increases after oral inoculation and then decreases until no oocysts are shed (Chapman, 1978; Matsui et al., 2006; Bangoura and Daugschies, 2007). It has been shown that oocysts shed in feces of infected hosts lose their infectivity after freezing at temperatures of less than approximately −10 °C (Landers, 1953; Lassen and Seppä-Lassila, 2014). In our experimental inoculations, the OPG transitions were similar (decreasing after the peak at 7–10 days PI) to those of other *Eimeria* spp., and after that time, no oocysts were detected in feces of some groups. In previous studies, *Eimeria* oocysts from Japanese rock ptarmigans survived after storage at 0 °C for 6 months (Matsubayashi et al., 2018a, Matsubayashi et al., 2018b); however, environment temperatures, including that of soils, can fall below 0 °C in the absence of snow (Sharratt et al., 1992). Thus, it is possible that oocysts present in the environment could be killed during winter. Indeed, no or only few oocysts as potential infection sources were detected in soils of the Japanese Alps in a previous study (Matsubayashi et al., 2021). To date, it remains unclear whether *Eimeria* oocysts can survive the winter, but the detection rate and OPG in Japanese rock ptarmigans have been shown to increase toward summer (August and September) (Matsubayashi et al., 2018b). Here, although only one chick was examined, all of the zoites had not disappeared, and a small number of parasites was still detected in the ileum at 37 days PI (Table 2) by histopathological analysis. Therefore, we speculate that *Eimeria* spp. can remain dormant in the birds during winter and begin proliferating again toward the summer. However, further studies are needed to determine how the parasites survive winter, as no data regarding *Eimeria* spp. infection of Japanese rock ptarmigans in winter are available.

It has been suggested that populations of rock ptarmigans isolated across multiple refugia diverged during the Wisconsin glaciation period and that geographic variations in subspecies reflect patterns of recolonization as glacial relicts after the ice receded (Holder et al., 1999). Consequently, a total of 31 subspecies have been described worldwide (Avibase, https://avibase.bsc-eoc.org/avibase.jsp). However, the origins and evolution of *Eimeria* spp. remain unknown. Interestingly, the weight gain of chicks inoculated with a lower dose of oocysts was greater than that of the controls, although the number of birds examined was low. Although further study is needed, our data suggest that mild infection with *Eimeria* spp. that results in acquired immunity might support chick growth and survival. We used Svalbard rock ptarmigans instead of Japanese rock ptarmigans in the present study. The pathogenicity of the parasites in the natural hosts, Japanese rock ptarmigans, and of infection with one species of *Eimeria* parasites, requires more extensive study. However, the possibility that *Eimeria* spp. adapted to glacial relict birds in addition to timber environments could coexist in isolated regions may open new areas for research in protozoan parasitology, but further studies are needed to confirm this possibility.

## Ethics statement

Fecal collection was performed in a non-invasive manner. In the experimental infection study, the animals were handled in accordance with the protocols approved by the Animal Care and Use Committee in accordance with the Animal Experimentation Guidelines of the Osaka Metropolitan University (approval numbers 15003 and 21022). No human samples were involved in this study.

## Declaration of competing interest

All the authors confirm that we declare that they have no conflict of interest.
